# An unusual cause of large bowel obstruction: To keep an open mind

**DOI:** 10.1002/ccr3.3292

**Published:** 2020-09-08

**Authors:** Tuhin Shah, Arjana Shakya

**Affiliations:** ^1^ Department of Surgery Asia Pacific Medical College Birgunj Nepal; ^2^ Department of Ophthalmology Asia Pacific Medical College Birgunj Nepal

**Keywords:** enterolithiasis, intestinal obstruction, large bowel obstruction, unusual

## Abstract

Enterolithiasis or formation of gastrointestinal concretions is an uncommon medical condition that develops in the setting of intestinal stasis due to various pathologies. Its prevalence ranges upto 10% and can present in different clinical pictures to challenge a clinician.

## Graphical abstract

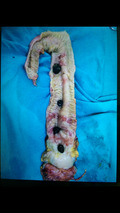

## 1 QUESTION

A 60‐year‐old lady presented with features of large bowel obstruction to the emergency room. She was admitted twice before with features of subacute bowel obstruction at another center but was managed conservatively. Her blood reports showed leucocytosis while the rest was normal. Her abdominal X‐ray showed few dilated ileal loops with multiple air fluid levels. Unusual finding on X‐ray was the presence of enteroliths on the right lumbar and hypochondriac region. What do you suggest should be the management?

- Conservative management
- Laparotomy and extraction of the enteroliths alone
- Colonoscopy
- Exploratory laparotomy with bowel resection without cholecystectomy
- Exploratory laparotomy with bowel resection with cholecystectomy

**FIGURE 1:**
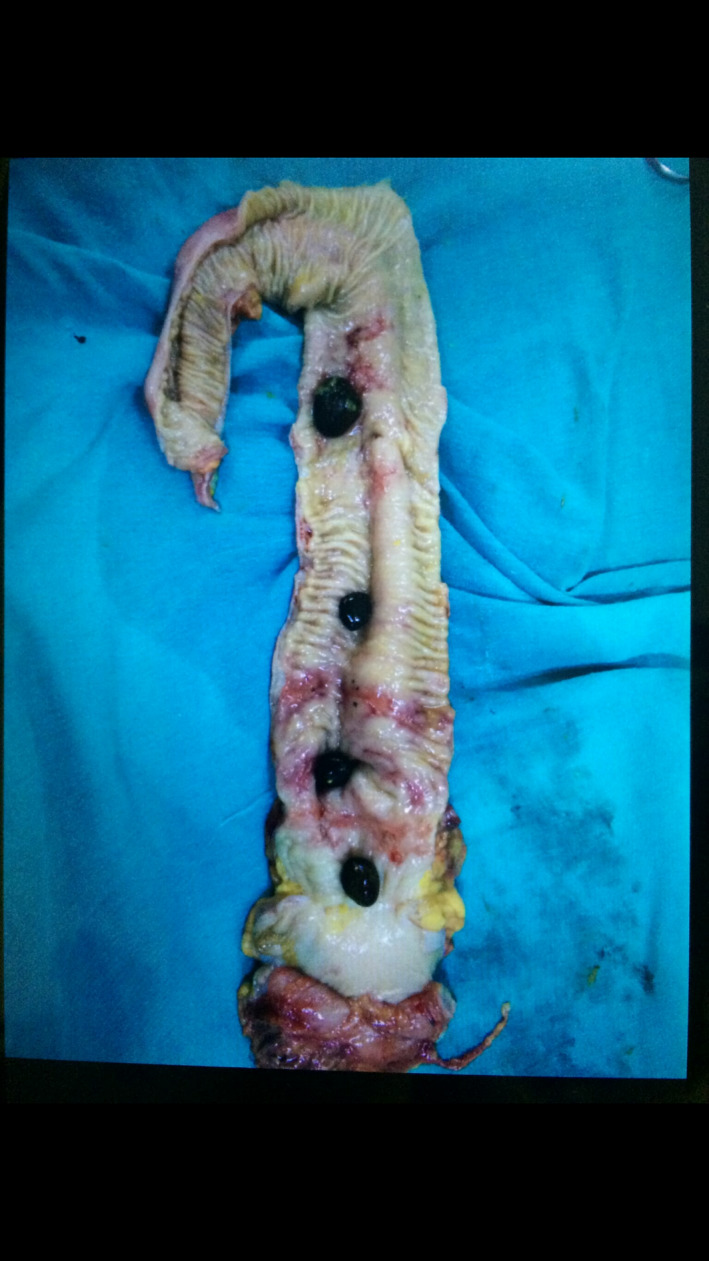
Right hemicolectomy specimen with the enteroliths within

Here in our case, we had to do an exploratory laparotomy with right hemicolectomy and cholecystectomy, as it was a case of gall stone ileus with stricture in the ascending colon suspicious of underlying pathology (Figure 1).

Enteroliths are classified as primary enteroliths—which are formed within the gastrointestinal tract or secondary enteroliths, stones which form outside the bowel and migrate to the intestine,^1^ of which the most common type is gallstones. Optimal treatment of enterolithiasis should focus on enterolith removal and correction of underlying pathology to prevent future formation of additional enteroliths.^2^

## CONFLICT OF INTEREST

None declared.

## AUTHOR CONTRIBUTIONS

Tuhin Shah: contributed to study conception and design, and acquisition of data; analyzed and interpreted the data; drafted the manuscript. Arjana Shakya: drafted the manuscript and performed critical analysis.
